# Malignant catarrhal fever in a goat: manifestation of virus-induced erythema multiforme

**DOI:** 10.1177/10406387231224906

**Published:** 2024-01-11

**Authors:** Grace Makanaka Makoni, Christian Gerspach, Nina Fischer, Giuliana Rosato, Rosalie Fabian, Paula Grest, Anja Kipar

**Affiliations:** Institute of Veterinary Pathology, Vetsuisse Faculty, University of Zurich, Zurich, Switzerland; Department of Farm Animals, Vetsuisse Faculty, University of Zurich, Zurich, Switzerland; Dermatology Unit, Clinic for Small Animal Internal Medicine, Vetsuisse Faculty, University of Zurich, Zurich, Switzerland; Institute of Veterinary Pathology, Vetsuisse Faculty, University of Zurich, Zurich, Switzerland; Institute of Veterinary Pathology, Vetsuisse Faculty, University of Zurich, Zurich, Switzerland; Institute of Veterinary Pathology, Vetsuisse Faculty, University of Zurich, Zurich, Switzerland; Institute of Veterinary Pathology, Vetsuisse Faculty, University of Zurich, Zurich, Switzerland. (Makoni, Rosato, Fabian, Grest, Kipar)

**Keywords:** arteritis, erythema multiforme, goats, malignant catarrhal fever, ovine herpesvirus 2, ulcerative dermatitis

## Abstract

Malignant catarrhal fever (MCF), caused by ovine herpesvirus 2 (OvHV2; *Orthoherpesviridae*, *Macavirus ovinegamma2*), has sheep as natural hosts. OvHV2 is an important macavirus globally that induces fatal disease in dead-end hosts. Goats, which can be infected subclinically with OvHV2, rarely develop MCF. A 28-wk-old female goat was presented with fever and multifocal crusty skin lesions. Histologic examination of a skin biopsy suggested erythema multiforme (EM), with pyoderma and dermal vasculitis. The doe was euthanized and subjected to postmortem and histologic examination. MCF was suspected and PCR assays for macaviruses were performed, followed by immunohistochemistry (IHC) for OvHV2 latency-associated nuclear antigen (oLANA), RNA in situ hybridization for Ov2.5 mRNA, and IHC to characterize infiltrating leukocytes. The main postmortem finding was severe multifocal ulcerative dermatitis with macrophage- and T cell–mediated arteritis. The latter was also detected in kidney, spleen, heart, and intestinal wall. The PCR assay detected high loads of OvHV2 in tissues. OvHV2 oLANA and Ov2.5 mRNA were expressed within the lesions in leukocytes, endothelial cells, fibroblasts, and/or keratinocytes. Our case confirms that MCF can initially manifest clinically as a skin disease in goats and as EM with confirmed viral etiology.

A 28-wk-old female goat (Bündner Strahlenziege, syn. Grisons Striped) was presented with fever and severe crusty-to-ulcerative lesions involving the oral mucosa, nasal planum (Fig. 1A), the inside of both pinnae, on teats, and in the interdigital spaces. Cytologic examination revealed pyogranulomatous inflammation with numerous intralesional coccoid bacteria. The main clinical differential diagnoses were contagious pustular dermatitis (orf), pemphigus foliaceus, zinc deficiency, and secondary pyoderma. Virologic examination (real-time PCR assay) excluded orf virus (ORFV; *Poxviridae*, *Parapoxvirus*) infection. The serum zinc concentration was reduced (385 µg/L; commercial laboratory RI: 650–1,250 µg/L).

**Figure 1.**
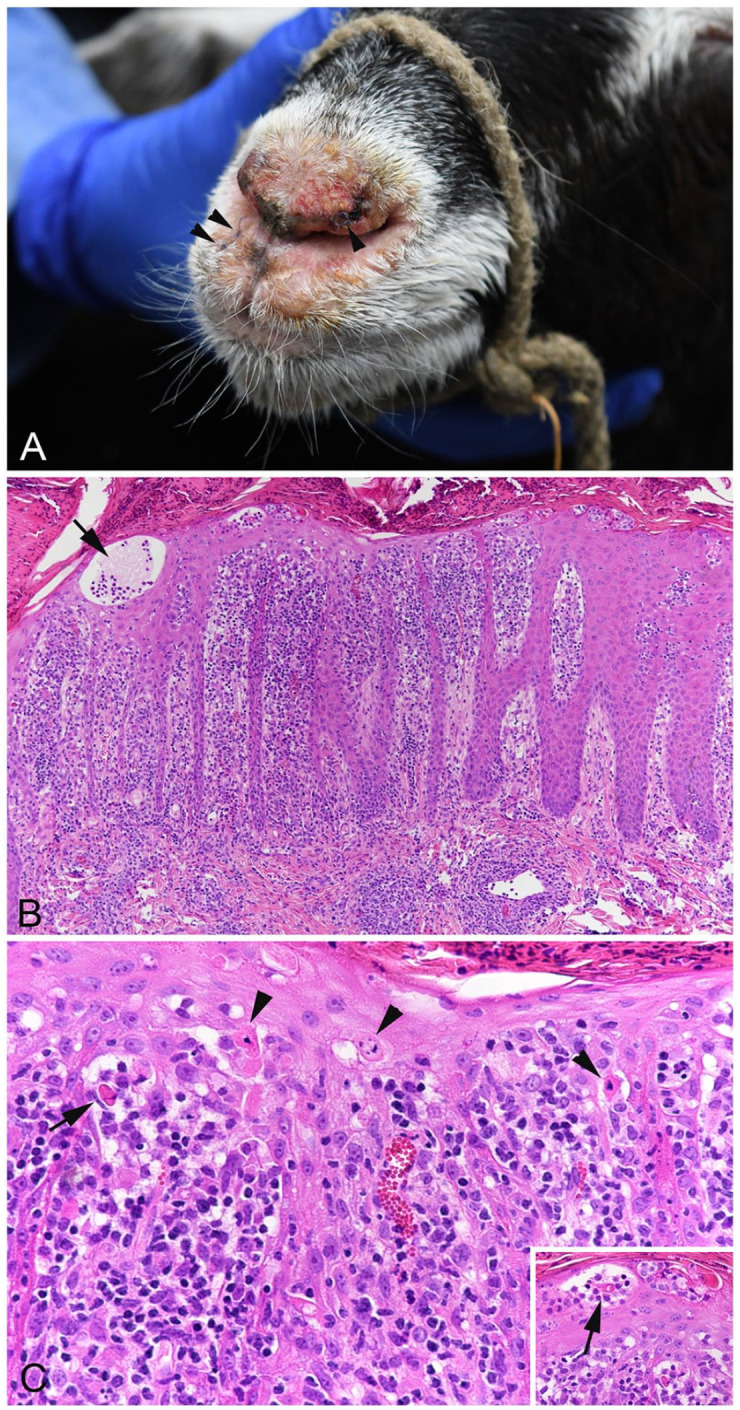
Goat with malignant catarrhal fever and virus-induced erythema multiforme. **A.** Clinical image of the nose (arrowheads: biopsy sites). **B, C.** Skin biopsy from the pinna showing severe dermatitis with intraepidermal pustule (B: arrow), apoptotic keratinocytes (C: arrowheads), and satellitosis (C inset: arrows). H&E.

Based on the cytologic findings, secondary bacterial dermatitis was suspected. After systemic pretreatment with antibiotics, skin biopsies were taken from the pinna and muzzle and submitted for histologic examination. The main histologic findings were transepidermal apoptosis with satellitosis, moderate interface dermatitis, severe lymphohistiocytic dermatitis with mild-to-moderate lymphohistiocytic vasculitis, and severe superficial pyoderma with intraepidermal and subcorneal neutrophil-dominated pustules, a few acantholytic keratinocytes, focal ulceration, and crust formation (Fig. 1B, 1C). PAS reaction did not reveal any fungal structures. The histologic changes were interpreted as most consistent with erythema multiforme (EM).^19^

Given the lack of distinct orthokeratotic and parakeratotic hyperkeratosis, zinc deficiency was discarded as a differential diagnosis.^8,12^ Epidermal proliferation and ballooning degeneration, both typical findings in contagious pustular dermatitis,^10^ were also not observed. The rare acantholytic intrapustular keratinocytes were interpreted as a consequence of pyoderma, and the dermal vasculitis, not a typical feature of EM, as a potential immune-mediated lesion of unknown origin.

EM has only been reported once in a goat; it was interpreted as an idiopathic form complicated by secondary bacterial folliculitis.^14^ In our case, viral infection, drug administration, or neoplasia were suggested as possible causes, considering that, in humans, both viral infections (most frequently herpesvirus) and drug reactions are common triggers for EM.^9^

Despite a week of intensive treatment with amoxicillin–clavulanic acid (Synulox; Zoetis), metronidazole (Minalgin; Streuli), and zinc, the animal’s general condition deteriorated. The goat became apathetic, developed dyspnea, and was euthanized 9 d after initial presentation.

Postmortem examination was undertaken with the owner’s consent. Crusty ulcerative skin lesions consistent with those reported clinically were found on lips, ears, nose, vulva, and teats (Fig. 2A, 2B). Similar skin lesions were also present, although to a lesser extent, on the back, between the scapulae. The nasal mucosa was covered by a moderate amount of yellow-to-green mucus. Gross changes were not evident in internal organs. Tissue samples were collected from lesions and all major organs and tissues, fixed in 10% neutral-buffered formalin, and processed routinely for histologic examination.

**Figure 2.**
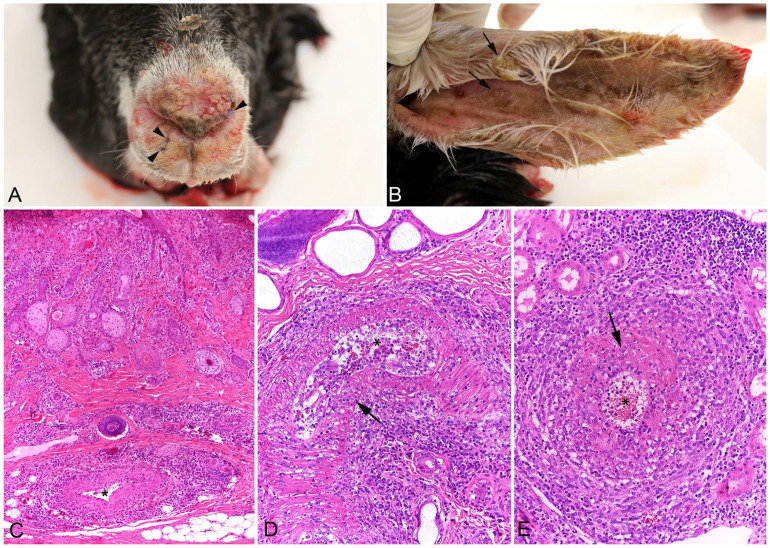
Goat with malignant catarrhal fever and virus-induced erythema multiforme. **A.** Autopsy: biopsy sites (arrowheads; 1 wk after sampling) on the nose. **B.** Crust formation in the pinna (arrows) at autopsy. **C.** The dermatitis at the ear is associated with mild mononuclear arteritis (*) and periarterial infiltration in the deep dermis. H&E. **D.** Dermal artery (*) at the nose biopsy with degenerative changes, focal leukocyte infiltrate in the media (arrow), and perivascular infiltration. **E.** Renal artery (*) with degeneration, leukocyte infiltration of the wall (arrow), and perivascular infiltration.

All of the lesions on the skin and nasal septum were histologically comparable to the biopsy specimens. However, the changes were more severe, with extensive ulceration, severe transepidermal apoptosis with satellitosis, interface dermatitis, and severe folliculitis with disruption of hair follicles. The underlying dermis often had severe multifocal mononuclear perivascular infiltrates and arteritis (Fig. 2C), sometimes with overt medial degeneration (Fig. 2D). Arteritis was also found in the kidneys (mainly larger arteries in the medulla and pelvis; Fig. 2E), spleen, heart (subendocardial artery), and intestinal submucosa. In immunohistochemistry (IHC), using cross-reactive antibodies against CD3 (T-cell marker; monoclonal mouse anti-human CD3, clone F7.2.38, Agilent Dako) and Iba1 (monocyte-macrophage marker; polyclonal rabbit anti-rat Iba1, Wako Chemicals),^16^ with consecutive negative control sections incubated without the primary antibodies or with a non-reactive mouse and rabbit antibody respectively, the inflammatory infiltrates were comprised predominantly of T cells and macrophages (Fig. 3A, 3B); occasional neutrophils were also observed.

**Figure 3.**
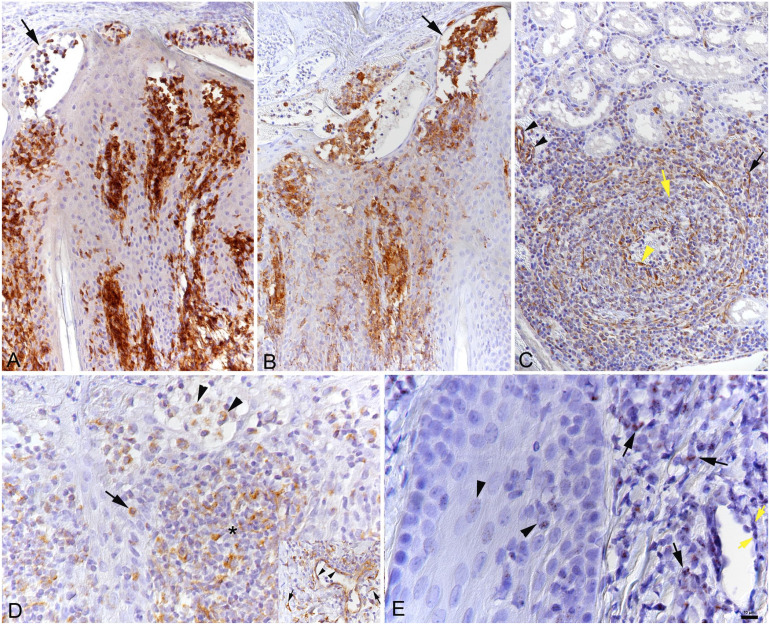
Goat with malignant catarrhal fever and virus-induced erythema multiforme. **A.** Abundant T lymphocytes in the inflammatory infiltrate and pustules (arrow) in the pinna skin biopsy. CD3 immunohistochemistry (IHC). **B.** Abundant macrophages in infiltrate and pustules (arrow) in the pinna skin biopsy. Iba1 IHC. **C.** Renal artery with viral antigen expression in leukocytes in the inflammatory infiltrate (yellow arrow), fibrocytes (black arrow), and vascular endothelial cells both in the affected artery (yellow arrowhead) and unaffected vessels (black arrowheads). OvHV2 latency-associated nuclear antigen (oLANA) IHC. **D.** In the skin biopsy from the pinna, viral antigen is detected within cells in the inflammatory infiltrate (*) and individual leukocytes in epidermis (arrow) and pustules (arrowheads). OvHV2 oLANA IHC. Inset: vascular endothelial cells (arrowheads) and fibrocytes (arrows) also express oLANA. **E.** Ov2.5 mRNA expression in the nucleus of infiltrating leukocytes (black arrows), keratinocytes (arrowheads), and vascular endothelial cells (yellow arrows) in the skin biopsy from the pinna. Ov2.5 mRNA in situ hybridization.

Further histologic changes were mild multifocal nonsuppurative leptomeningitis and encephalitis, moderate multifocal lymphocyte-dominated (peri)bronchial and perivascular infiltration in the lung, mild diffuse lymphocyte-dominated mononuclear enterocolitis, mild-to-moderate multifocal lymphocyte-dominated mononuclear portal infiltration and hepatocellular lipidosis, and mild focal mononuclear interstitial pancreatitis. The spleen had mild depletion of follicles and T-cell zones.

Given that systemic mononuclear arteritis was the dominant pathologic process^18^ and realizing that dermatitis has been reported as a feature of OvHV2-induced malignant catarrhal fever (MCF) in goats,^2,5^ we thus suspected MCF. Therefore, tissue samples that had been collected from lung, kidney, and lesioned skin during autopsy were subjected to a quantitative real-time PCR (qPCR) assay for gammaherpesviruses (OvHV2, OvHV1, CpHV2, BoHV6) as described previously.^15,16^ We found infection with OvHV2 and very high viral copy numbers in all tested organs (skin: 37,091/100 ng DNA; kidney: 55,961/100 ng DNA; lung: 207,312/100 ng DNA), but did not detect any other gammaherpesvirus.

Having confirmed the causative agent, we attempted to identify its target cells. We subjected consecutive sections of affected tissues to IHC for the OvHV2 latency-associated nuclear antigen (oLANA), using a custom polyclonal rabbit antibody and following a published protocol.^16^ RNA in situ hybridization was performed on sections from the initial skin biopsies (RNAscope ISH method, automated RNAscope 2.5 detection reagent kit [brown]; Advanced Cell Diagnostics [ACD]) following the manufacturer’s instructions and an established protocol,^16^ with slight modifications. We observed strong expression of OvHV2 oLANA in infiltrating leukocytes, endothelial cells in affected arteries, dermal fibroblasts, and endothelial cells of unaffected vessels (Fig. 3C, 3D); Ov2.5 mRNA was expressed in the nuclei of infiltrating leukocytes, keratinocytes, and vascular endothelial cells in the skin biopsy (Fig. 3E).

MCF is a rare disease in goats. Authors of a 2004 review stated that, although goats can be infected with OvHV2, they do not develop clinical MCF under natural conditions.^3^ This assumption was first proved incorrect in 2006 when a case of confirmed OvHV2 infection was reported in a pygmy goat.^18^ Another 2006 publication referenced a suspected case in a domestic goat, as part of an outbreak affecting several ruminant species in a zoologic garden in 1973; however, this case was not confirmed by histology or virology.^18^ In 2007, 3 natural MCF cases were reported in goats,^7^ followed in 2010 by a case in an 18-mo-old crossbred doe^5^ and, in 2014, an 8-mo-old female dwarf goat.^2^ The reports mention a plethora of clinical signs, such as pyrexia, diarrhea, generalized lymph node enlargement, cloudy eyes, and neurologic signs.^7,18^ Pathologic findings were overall similar; arteritis with fibrinoid degeneration of the vessel wall, as well as mononuclear pericholangitis, interstitial nephritis, meningitis, and perivascular infiltrates in the brain, have been reported previously.^2,18^ These pathologic changes were similar to those reported in cattle^13^; the vasculitis in our case was composed of the same inflammatory infiltrate, and virus was present in the lesions.^16^

However, our case is of particular interest because of the skin lesions. At the time of clinical presentation, when ORFV infection had been excluded, the skin lesions were most consistent with EM, a condition that is thought to represent a cell-mediated immune response against certain antigens, resulting in cytotoxic interface dermatitis, with apoptotic keratinocytes.^19^ In humans, EM is most frequently associated with herpes simplex virus infection and is thought to be directed against viral antigens deposited in lesional skin.^1^ In our case, we found viral (Ov2.5, encoding viral IL-10) mRNA expressed in keratinocytes, which would indicate a pathogenesis similar to that of human EM, with an infiltrate dominated by T cells and macrophages.^1^ Cutaneous lesions are observed in cattle with MCF, reported as crusting and ulcerative processes,^11^ and in Sika deer^4,6^; such lesions have also been reported twice in goats.^2,5^ To date, the lesions have not been considered as consistent with or interpreted as EM.

Upon initial presentation, dermal vasculitis was noted but was not a dominant feature in our case. One week later, at the time of euthanasia, however, the arteritis was severe and was confirmed to be OvHV2-associated. Viral oLANA and mRNA were not only detected in the infiltrating leukocytes (T cells and macrophages) but also in vascular endothelial cells, with high viral loads in affected tissues. This suggests a virus-driven pathogenesis for both the vasculitis and dermatitis. Interestingly, there have been 2 reports in which OvHV2 DNA was detected in the skin, one in cattle^11^ and the other in a free-ranging bighorn sheep (*Ovis canadensis*).^17^ The latter had multifocal granulomatous and eosinophilic mural folliculitis, apoptotic keratinocytes, satellitosis, and intracorneal pustules. Viral DNA copy numbers were approximately one-tenth those in our case (4,120 copies/100 ng DNA^17^ vs. 37,091 copies/100 ng DNA). The authors concluded that the virus “played a key role in the development of the chronic dermatitis.” Our results indicate that MCF can be associated with EM; it might be useful to (re-)examine skin lesions in MCF cases of other species to further substantiate or discard this hypothesis.
